# User preferences for coworking spaces; a comparison between the Netherlands, Germany and the Czech Republic

**DOI:** 10.1007/s11846-020-00414-z

**Published:** 2020-09-17

**Authors:** Rianne Appel-Meulenbroek, Minou Weijs-Perrée, Marko Orel, Felix Gauger, Andreas Pfnür

**Affiliations:** 1 Urban Systems & Real Estate, Eindhoven University of Technology, Eindhoven, The Netherlands; 2 University of Economics in Prague, Prague, Czech Republic; 3 Technical University of Darmstadt, Darmstadt, Germany

**Keywords:** Coworking spaces, MMNL, User preferences, Space attributes, Workplace, D16, M14, O52, O57, R30, P25

## Abstract

Coworking spaces have become a central component of new work environments, with large international chains. The purpose of this study is to investigate whether user preferences for the physical workspace design are consistent across countries, which the uniformity of such chains seems to suggest. A comparison between the user preferences of coworking spaces between the Netherlands (n = 219), Germany (n = 98) and the Czech Republic (n = 79) is performed using a mixed multinomial logic model for each country. Besides statistical utility of attributes, also motivations for working in coworking spaces are analysed. The findings show that there are some consistencies in preferences across countries. Typical real estate characteristics like accessibility and contract options came forward to be the most important attributes in choosing which coworking space to work at in all three countries. However, significant differences in the desired quality levels of specifically these attributes were found between the countries as well, and only the less important attributes showed similar preferences internationally. This suggests that identical world-wide implementations of the same concept, might serve multi-nationals but possibly will not attract local users. The identified differences in preferences can help to position more specific, dedicated coworking spaces within local markets.

## Introduction

For the last two decades, work practices have changed due to the collaborative economy and new forms of collaboration (Mitev et al. 2019). One of the consequences is the continuing rise of coworking spaces. While the contemporary versions of these collaborative and shared workplaces started to gain visibility around the year 2005 in San Francisco (The Spiral Muse) and London (The Hub) (Merkel 2015; Waters-Lynch et al. 2016), their existence can be tracked to the mid-90 s (Orel and Dvouletý 2020). Nonetheless, Deskmag’s yearly global coworking survey (Deskmag 2018) showed that 29% of all coworking spaces available in 2018 were opened over the last year. Up until 2022, the number of coworking spaces is expected to grow at an annual rate of 6% in the U.S. and 13% elsewhere (pre-Covid-19 expectations).

This new model of working alone-together (Spinuzzi 2012) is not only elusive to practitioners but also increasingly an intrigue of academics (Waters-Lynch et al. 2016). Based on the concept of the urban sociologist Ray Oldenburg who coined the term *third places* (Oldenburg 1989), several authors deal with the classification of coworking spaces (Bouncken et al. 2018; Merkel 2015). Coworking spaces have been studied extensively in the USA (Spinuzzi 2012), different European countries (Bouncken et al. 2018; Gerdenitsch et al. 2016; Rus and Orel 2015; Thierstein and Marx 2016; Weijs-Perrée et al. 2019), Asia (Bouncken et al. 2016; Soerjoatmodjo et al. 2015), and Australia (Butcher 2013; Waters-Lynch and Potts 2017). These studies are related to many different theories and extant literature, mainly based on the disciplines of management, psychology/sociology and economics. However, they generally lack a clear insight in the user preferences for the physical workspace design and especially whether this differs between countries, because of their single country focus. Bouncken et al. (2016; p319-320) state that: “So far it is unclear how such spaces should be set up, […] and which business models suit the users and providers of coworking-spaces”. Even if the decision of using coworking spaces is done by the management of a company, the extent to which coworking space benefits suit end-users heavily relies on their expectations and preferences. The workplace support of end-users influences several organizational outcomes such as productivity and collaboration (Appel-Meulenbroek et al. 2018).

From an operator’s view, user preferences can serve as a foundation for value propositions of coworking business models (Clauss et al. 2019). Attracting new members has been and remains the number one challenge for operators (Deskmag 2018). However, as Bouncken et al. (2016) mentioned, there is limited understanding of how coworking space operators can design their business models for differing user demands. As Yang et al. (2019) state, coworking space users prefer different services and spaces. Local, small coworking operators are specializing in specific local user groups and show a high diversity in terms of strategy, location and set-up (Bouncken et al. 2020a). At the same time, several coworking operators have started providing nearly identical looking coworking spaces worldwide (e.g. WeWork, Impacthub, Spaces) to cater multi-nationals. As a result, Bouncken et al. (2020a) call for the identification of global success factors of coworking spaces. As research has not yet identified whether user preferences for coworking spaces are consistent across countries (Bueno et al. 2018; Mitev et al. 2019), it remains unclear whether the international models are able to cater multi-national and local needs. Therefore, this paper aims to answer the research question: Are user preferences consistent among coworking space users across different countries? The main contribution of this paper is its international comparison of user preferences and motivations, identifying which are consistent and which change regionally. Data were gathered among users of several coworking spaces in three different countries, namely the Netherlands (Western Europe), Germany (Western Europe) and the Czech Republic (Eastern Europe), to test hypotheses on assumed consistency in preferences across these countries.

After a literature review of the coworking phenomenon and its users, the most important space characteristics were drawn from literature for a stated choice experiment. A stated choice based questionnaire among 219 coworkers from the Netherlands, 98 coworkers from Germany and 78 coworkers from the Czech Republic (396 coworkers in total) was performed to identify preferences for hypothetical coworking spaces described by their most relevant characteristics. Multinomial logit modelling was used to analyse the preferences for coworking space characteristics and to see whether the countries show differences in user preferences regarding accessibility, atmosphere, layout, diversity of spaces and of tenants, services and contracts. The research has practical implications for corporates, individual user and operators and enhances theoretical understanding of coworking spaces as institutions in an entrepreneurial ecosystem.

## Theoretical background

Every operational activity requires not only a distribution of tasks to the personnel resources, but always also a spatial organisation of the task completion in workplaces, rooms, buildings and locations (Krüger 1984). Whereas in larger companies the task of the spatial organisation of work is usually performed by a separate department known as corporate real estate management (Brown et al. 1994), in smaller organisations this task is performed by the management itself. The core task in both cases is to manage the planning, provision, use, operation and exploitation of workspace. The provision of office space is a secondary process that optimally supports the core process of a company. Kämpf-Dern and Pfnür (2014) show that in corporate practice there is no one-best model, but there is a best fit. Coworking spaces are an innovative alternative form of space provision to the traditional provision forms of office space. From the point of view of the organisation, the use of coworking spaces is a sourcing decision. Companies must consider whether it makes more sense and is more efficient to provide internal services or to use external services for their spatial organisation of work.

Nowadays, the users of coworking spaces include a wide range of actors as predicted by Sargent et al. (2018). Larger companies show increased propensity to support their employees and their tendencies for work on a dislocated, telework basis (Walden 2019) to blend in with independent workers and their networks (Leclercq-Vandelannoitte and Isaac 2016). Gauger et al. (2020) analysed that particularly in mature coworking markets, start-ups are almost being crowded out by users from large enterprises. Small-to-mid sized enterprises pursue the usage of coworking spaces to allow cost savings through the use of shared office facilities, and to interconnect their employees in various collaborative networks (Arora 2017). Other users include independent workers such as freelancing individuals, seniors, unemployed and others (Mitev et al. 2019).

### Benefits of coworking

By bundling all real estate services into a space-as-a-service package, coworking providers offer external service provision. In the entrepreneurship literature, this space-as-a-service concept is considered as one component of an entrepreneurial ecosystem. Coworking spaces as the spatial entity of an entrepreneurial ecosystem can add value by knowledge management and networking opportunities. Bouncken et al. (2018) term coworking spaces within the framework of institutional theory. Coworking spaces can be regarded as (prototype) institutions for entrepreneurship and innovation, that not only create and appropriate value, but also give space for tensions (Bouncken et al. 2018, 2020a). Fuzi (2015) describes these spaces as environments that facilitate social support, innovation, creativity, knowledge sharing and collaboration. Besides attracting people with different profiles and social interactions being central to the concept, coworking spaces also enhance productivity (Bueno et al. 2018), stimulate knowledge transfer among coworkers (Kopplin 2020), and create a working community (Weijs-Perrée et al. 2016) that can be perceived as a source of social support for coworking space users leading to higher quality and satisfaction of work (Gerdenitsch et al. 2016).

Not all end-users have the same motivations for choosing a coworking space though. Spinuzzi (2012) found that coworkers seek many benefits from using a coworking space related to interaction, feedback, trust, learning, partnerships, encouragement and referrals. The gathering of users in coworking spaces strategically increases the likelihood of unpredictable encounters and offers the possibility of "social learning". Fabbri (2015) adds that belonging to a coworking space can also increase credibility and access to partners through a ‘labelling’ and ‘window’ effect. Bouncken et al. (2016) divided the sought benefits of coworking users in two business models, namely those seeking for efficiency and those seeking for novelty. Regarding efficiency, existing studies have mentioned motivations to use a coworking space such as affordable accommodation (Fuzi 2015; Merkel 2015), the professional appearance for the company (Fabbri 2015), a professional supportive work environment (Spreitzer et al. 2015), looking for a workplace outside the home/separating work and private life (Fuzi 2015) and flexibility regarding rental period and number of square meters (Sykes 2014). Regarding novelty, motivations that come forward from existing studies are the feeling of being part of a community (Spreitzer et al. 2015), the vibrant and creative atmosphere in the coworking space (Merkel 2015), the opportunity to network with coworkers (Fuzi 2015) and to have social- (Merkel 2015) and work-related interactions (Fuzi 2015). But the main added value of a coworking space is suggested not to be a favorable rent or a more pleasant working environment than home, but the possibility of collaborating with other coworkers when ideas, resources and necessary information are lacking (Waters-Lynch and Potts 2017). Therefore, we pose the following hypothesis:

H1: Novelty based motivations to work at a coworking space are more important than those based on efficiency.

As there is no previous research comparing such motivations across countries, the apparently successful ‘uniformity approach’ by large international coworking chains is used to pose the next hypothesis:

H2: The ranking of motivations to work at a coworking space is consistent across countries.

### Coworking space attributes

To study preferences of users, it is important to identify the most important attributes of coworking spaces that can satisfy or frustrate certain user preferences. Although many hybrids between coworking spaces and other business centre concepts are increasingly present, Waters-Lynch et al. (2016) point out differences from other shared office concepts in the aesthetic design of coworking spaces. Weijs-Perrée et al. (2016) also showed a clear difference with serviced offices, which are all located in modern office spaces, while coworking spaces can also be located in former industrial era warehouses or factories (Deskmag 2016) and are thus more diverse in outside and inside appearance (Bouncken et al. 2020b).

First, like many offices, most coworking spaces are positioned in highly accessible locations, but more ‘remote’ spaces exist as well (Bouncken et al. 2020a). Generally, accessibility by car and/or by public transport can be distinguished as relevant attribute levels. Regardless of their location, as a second distinguishing attribute coworking spaces can be located in new, modern offices but also in former industrial era warehouses (Deskmag 2016) with more complex structural elements (Gertner and Mack 2017). Early coworking spaces made use of practical furniture when setting-up typical home-like environments with couches, kitchen desks and other home-based furniture (Brown 2017; Neuberg 2005). In regard to that, it could be emancipated that early coworking environments have had a very different atmosphere and aesthetics in contrast to modern coworking centres that tended to incorporate social and emotional meanings within the spatial design of the workspace that could positively affect users (Bouncken et al. 2020c). A third attribute is the layout of the space, as openness has been shown to influence face-to-face interactions in offices (Rashid et al. 2006) and thus possibly how the social component is experienced (Bouncken et al. 2020a). Generally, coworking spaces have an open layout (Gertner and Mack 2017), as this is likely to stimulate interaction between coworkers as intended, but a half-open layout can also be present and even individual, closed spaces are offered in some coworking spaces (Deskmag 2016) to cater all possible preferences (Wright 2018).

Besides the regular workspace, the majority of coworking spaces are combined with private meeting rooms and a kitchenette. However, the trend is progressively shifting in a way that many contemporary buildings offer additional types of spaces, such as informal break out zones and spaces for specific events (Deskmag 2016; Kojo and Nenonen 2016) and provide their users additional leisure and well-being services such as recreational facilities (e.g., gym, spa, etc.) and guided sport activities (e.g., yoga, meditation classes, etc.) (Cabral and van Winden 2020; Spinuzzi 2012). Coworking space providers principally tend to construct value capturing strategies and shape various offerings to introduce efficiency-centred, development-centred and user-centred coworking environments (Bouncken et al., 2020d). Especially the latter, user-centred form of a coworking space commonly integrates the position of a mediator or community manager who curates the interpersonal interactions and interconnects regular users in supportive networks (Merkel 2015; Rus and Orel 2015; Spinuzzi et al. 2019) with the aim of establishing collective action amongst coworking space users (Blagoev et al., 2019). Mediation activities are integrated through either spatial mechanisms (Bouncken and Aslam 2019), digital tools (Kopplin 2020), or actively used in the forms of communal events that promote interaction and participation in selected undertakings (Cheah and Ho 2019; Parrino 2015; Waters-Lynch and Potts 2017). According to Irving et al. (2020), the expected benefits of spatial interventions frequently fail to materialize, making human intervention a considerable factor when accelerating the rate of formal or informal interactions and knitting collaborative relationships. By that, community managers regularly play an essential role in coworking space development and positioning on the market (Bouncken et al. 2018; Gauger et al. 2020; Gregg and Lodato 2018). Depending on the business model behind the selected coworking spaces as well as the capacity of mediation mechanisms, Kojo and Nenonen (2016) separate coworking spaces in the category of third places, public offices, collaboration hubs, coworking hotels, incubators and shared studios. Despite the classification, all coworking spaces are characterised by array of diverse user-base with individuals having different professional and personal backgrounds.

With that in mind, the last two attributes concern the diversity of tenants and the lease contract. The description of coworking spaces by Parrino (2015) emphasized a diversity of users regarding their sector, although some coworking spaces focus on a specific sector. According to multiple studies (Fuzi 2015; Spinuzzi 2012; Sykes 2014), a short contract is also a typical coworking space characteristic. Some even have no contract at all, but longer contracts can also be an option (Durante and Turvani 2018). The following hypothesis is posed:

H3: Accessibility, atmosphere, layout, diversity in spaces, hospitality, events, diversity in tenants and the lease contract are significant attributes when choosing between different coworking space options.

As there is no previous research comparing user preferences for these attributes across countries, the apparently successful ‘uniformity approach’ by large international coworking chains is used to pose the last hypothesis:

H4: User preferences for coworking space attributes are consistent across different countries.

## Methodology

### Data collection

**Fig. 1:**
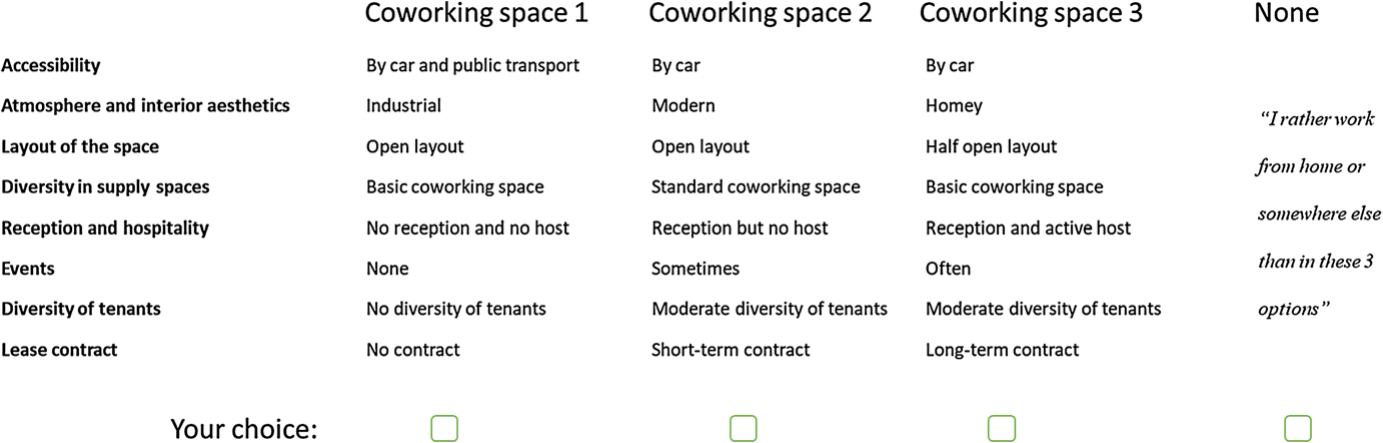
Example of a choice set in the stated choice part of the questionnaire

Data were collected with an online questionnaire consisting of two parts. First, respondents were asked to indicate their top 3 of motivations to work at a coworking space from a pre-determined list (to test H1 and H2). They also had to answer personal questions (gender, age, education level) and indicate work-related characteristics (user group, position within the organization, sector of the organization, and number of hours working at the coworking space). The second part of the questionnaire was setup as a stated-choice experiment (see Hensher et al. 2015) to be able to test H3-H4 on preferences for the coworking space attributes. In stated choice experiments, respondents chose between hypothetical alternatives, which are described by the quality levels for a list of relevant attributes for the offer that is studied. In this case, that ‘offer’ was a coworking space. Respondents were asked to choose between 3 different alternatives, plus they were offered the option to choose to rather work from home or at another location/coworking space than those alternatives that were presented. The alternatives varied based on the possible quality levels of the identified coworking space attributes from literature (see Table 1). Figure 1 shows an example of such a choice set.

**Table 1:** Atrributes and their levels

Attribute	Attribute level
Accessibility	Level 0: By car and public transport
	Level 1: By car
	Level 2: By public transport
Atmosphere and interior aesthetics	Level 0: Industrial
	Level 1: Modern
	Level 2: Homey
Layout of the space	Level 0: Open layout
	Level 1: Half open layout
	Level 2: Closed layout
Diversity in supply spaces	Level 0: Basic coworking space
	Level 1: Standard coworking space
	Level 2: Premium coworking space
Reception and hospitality	Level 0: No reception and no host
	Level 1: Reception but no host
	Level 2: Reception and active host
Events	Level 0: None
	Level 1: Sometimes
	Level 2: Often
Diversity of tenants	Level 0: No diversity of tenants
	Level 1: Moderate diversity of tenants
	Level 2: Strong diversity of tenants
Lease contract	Level 0: No contract
	Level 1: Short-term contract
	Level 2: Long-term contract

As each attribute has three levels, asking respondents to choose between each potential configuration of the attributes’ quality levels would result in (3^8^ =) 6561 possible alternatives. Therefore, an orthogonal fraction of this design was constructed (Hensher et al. 2015), consisting of 27 alternatives. These alternative spaces were randomly divided over 9 choice sets, with each 3 alternatives. So, each respondent thus evaluated all hypothetical workspaces (meaning a complete design) in only 9 questions.

The survey was spread in the Netherlands, Germany and the Czech Republic, within a period of three years (2016–2019). Throughout Germany and the Netherlands, the samples were collected in many different regions/cities and coworking spaces to provide a spatial heterogeneity of the participants. In Berlin, for example, there is a very mature coworking space market, which attracts many users from the IT sector, while in Frankfurt many users come from the financial sector. Furthermore, the survey was conducted in small and medium-sized cities, so that a cross-section of coworking spaces was achieved.

In Czech Republic, data was collected only in the main capital Prague. The city of Prague has been selected due to its geographic position and current situation of the coworking industry. Centrally positioned and with a midsized urban area, Prague has seen a swift growth of coworking spaces and users in recent years (Šindelářová and Kubikova 2018). In order to have a diverse sample, five different coworking spaces were selected, ranging from student-style coworking cafes to corporate coworking spaces.

The coworking spaces in all countries were visited personally to achieve a high response rate. In the Netherlands, a total of 219 respondents successfully completed the questionnaire, in Germany 98 respondents, and in Czech Republic 79 respondents.

### Analytic strategy

To study the assumptions on motivations (H1 and H2), first the rankings were studied themselves, after which the top 3 choices were added for each possible motivation per country to test H2 with a Chi-square analysis.

To study user preferences (H3) and their consistency across countries (H4), a mixed multinomial logit model (MMNL) for each country was estimated. A MMNL is a very efficient and flexible discrete choice model (Hensher et al. 2015; McFadden and Train 2000) for analysing data with a panel structure (i.e. multiple choices by the same respondent). Furthermore, using this approach, it is possible to capture unobserved heterogeneity (Train 2009). It estimates a constant utility parameter that reflects the alternative ‘none of these options’ in which a respondent rather would work at home or somewhere else than in one of the three hypothetical coworking spaces. The equation for the utility for user *i* for coworking space *j* on choice occasion *t* is:

$$ U_{ijt} = {\mathbf{\beta^{\prime}X}}_{ijt} + \varepsilon_{ijt} $$

where *X*_*ijt*_ represents all attributes of the coworking space with relative weights (parameters *β*) to be estimated. The error term, *ε*_*ijt*_, represents unobserved heterogeneity. This equation assumes that all the estimated parameters are equal for all users. Since the parameters relating to different levels of the same attribute are conceptually interrelated, a random parameter is estimated for only one of the parameters related to the same attribute to capture possible heterogeneity related to that attribute. As a somewhat arbitrary choice, the random parameters in the model relate to the significant, first levels of the attributes. A normal distribution is assumed for each random parameter. The non-random parameters in the model represent the second levels, plus non-significant first levels of the attributes. To estimate the parameters of the model, 1000 Halton draws were used (Bhat 2001).

## Results

### Sample description

Table 2 shows the characteristics of the samples from the Netherlands, Germany and the Czech Republic. The samples consist of a larger share of men with 52% in the Czech Republic, 57% in Germany and even 68% in the Netherlands, comparable to Deskmag’s (2018) worldwide statistics. The average age in the countries is 29 (SD = 6.8) in Czech Republic, 33 (SD = 9.4) in Germany and 35 (SD = 11.2) in the Netherlands, so a bit younger than Deskmag’s average of 36 years. The higher share of students (38%) in the Czech Republic sample explains the lower average age in this sample. Education levels of the users are generally high. On average the respondents worked 22 h per week in a coworking space.

**Table 2:** Sample characteristics

	Netherlands (N = 219)			Germany (N = 98)			Czech Republic (N = 79)			Total sample (N = 396)
	%	Mean	SD	%	Mean	SD	%	Mean	SD	%/ Mean
*Gender*										
Male	68			57			52			62
Female	32			43			48			38
*Age*		34.6	11.2		33.0	9.4		28.8	6.8	33.0
≤ 24 years	16			12			32			20
25–34 years	39			64			53			52
35–44 years	25			13			13			17
≥ 45 years	20			10			2			11
*Education level*										
Low education (i.e. secondary vocational education, pre-university education and intermediate vocational education)	14			8			0			9
High education (i.e. higher vocational education, university bachelor, master, PhD)	86			92			100			91
*Hours working at coworking space*		21.3	14.3		23.9	15.5		19.1	10.8	22.4
*Position in organization*										
Supporting staff	3			3			1			2
Regular employee	22			52			24			32
Manager	8			12			1			8
Board/owner	42			10			20			26
Does not apply	25			23			53			32
*User type*										
Self-employed worker, freelancer or entrepreneur	54			31			35			44
Employee of company (2–10 employees)	18			18			17			17
Employee of company (11 or more employees)	18			33			10			20
Student	12			18			38			19
*Sector*										
Consultancy (legal advice, organizational advice etc.)	25			14			6			19
Design (art, graphic, web, product, games etc.)	12			8			9			10
IT (software engineer, web developer etc.)	21			11			19			18
Education (coaching, training, teaching, etc.)	9			10			4			8
Research (scientist, analyst, researcher, etc.)	5			9			3			6
Project management, PR, marketing, sales, advertising, communication	8			12			15			9
Other sector	20			36			44			30

In all countries, many coworkers are self-employed workers (ranging from 31–54%). In Germany, many respondents (33%) work for a company with more than 11 employees and over half is a regular employee, while in the Netherlands 42% is owner/board member of their organisation. This could also be related to the fact that the hours spent in a coworking space is the highest in Germany (approximately 24 h, versus 21 in the Netherlands and 19 in the Czech Republic). With regard to sector, in the sample of the Netherlands coworkers are more frequently working in the consultancy sector compared to coworkers from Germany or the Czech Republic.

### Main motivations

**Fig. 2:**
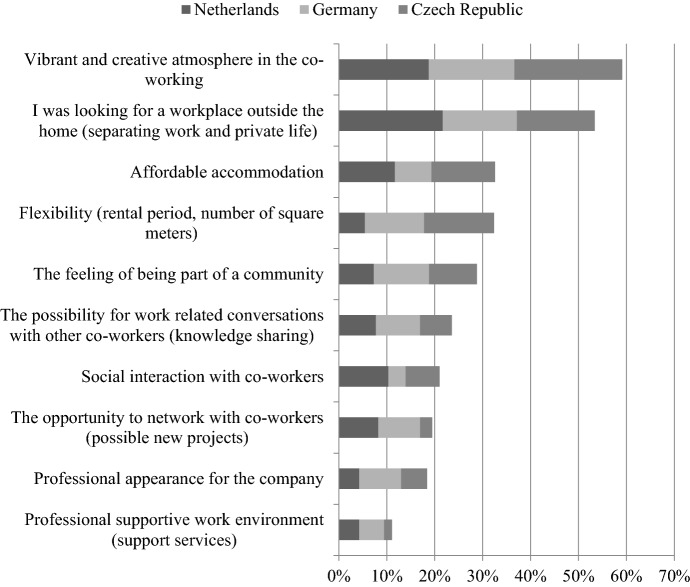
Main motivations per country to work at a coworking space

Figure 2 shows the main motivations per country to work at a coworking space. The percentages show the total share of choosing three main motivations to work at a coworking space. Overall, most respondents mentioned the vibrant and creative atmosphere as one of their main three motivations, which is a novelty-focused motivation. However, this was followed by several efficiency-focused motivations, such as separating work-and private life, affordable accommodation, and flexibility. Then, again novelty-focused motivations came forward regarding community and interactions. The bottom of the list also showed efficiency-focused motivations, regarding a professional, supportive work environment and appearance. So H1 (novelty based motivations to work at a coworking space are more important than those based on efficiency) is not very clearly confirmed. Although a vibrant/creative atmosphere is top of the list, it is immediately followed by several efficiency-focused motivations.

As can be seen in Fig. 2, there are differences between countries. For example, respondents from the Netherlands, choose “I was looking for a workplace outside the home” as the most crucial motivation to work at a coworking space, while in Germany and the Czech Republic this was the novelty-focused “vibrant and creative atmosphere”. A chi-square test shows that differences in the top 3 of motivations are significant (F(20,1172) = 69.935, *p* ≤ 0.000), so H2 (The ranking of motivations to work at a coworking space is consistent across countries) must be rejected. Both some novelty-focused motivations and some efficiency-focused motivations show particularly large differences between the observed and expected values. Regarding efficiency-focused motivations, respondents from the Netherlands more often chose flexibility (in rental period and/or number of square meters) in their top 3 motivations than in Germany and the Czech Republic. German coworkers particularly seemed to select professional appearance for the company and affordable accommodation more often, while in the Czech Republic the supportive services of a professional supportive work environment were selected relatively more often. Regarding novelty-focused motivations, the coworkers from the Netherlands relatively less often put social interaction with coworkers in their top 3, while Germans selected this relatively more often. Those from the Czech Republic relatively more often mentioned the opportunity to network with coworkers towards possible new projects.

### User preferences of the countries

Table 3 shows the results from the three MMNL models of the different countries. As can be seen, based on the samples of the three countries, one or more levels of all attributes were found to be significant. This finding suggests that all attributes are important for choosing a coworking space and thus H3 (Accessibility, atmosphere, layout, diversity in spaces, hospitality, events, diversity in tenants and the lease contract are significant attributes when choosing between different coworking space options) is accepted.

**Table 3:** Results MMNL country models

		Netherlands (N = 219)	Germany (N = 98)	Czech Republic (N = 79)
Attributes	Attribute level	Coefficient	Coefficient	Coefficient
**Random parameters**				
Constant	Constant	1.3388***	1.1891***	− 0.3915
*Accessibility *	*By car and public transport*	0.5949***	0.5770***	0.9137***
Atmosphere and interior aesthetics	Industrial	− 0.1587***	− 0.2869**	0.0190
Layout of the space	Open layout	0.0551	0.0424	0.3010**
Diversity in supply spaces	Basic coworking space	− 0.0595	− 0.1692**	− 0.0180
Reception and hospitality	No reception and no host	− 0.2109***	− 0.3542***	− 0.1008
Events	None	− 0.1727***	− 0.1334*	− 0.2315**
Diversity of tenants	No diversity of tenants	− 0.3239***	− 0.4287***	− 0.6606***
*Type of lease contract*	*No contract*	0.6638***	0.1683	0.4671***
**Non-random parameters**				
*Accessibility*	By car	− 0.6118***	− 0.8506***	− 1.9389***
Reference level	*By public transport*	0.0169	0.2736	*1.0252*
*Atmosphere and interior aesthetics*	*Modern*	− 0.004	0.1590*	− 0.1785*
Reference level	*Homey*	*0.1627*	0.1279	*0.1595*
*Layout of the space*	*Half open layout*	0.3200***	0.4508***	0.3918***
Reference level	Closed layout	− 0.3751	− 0.4932	− 0.6928
*Diversity in supply spaces*	*Standard coworking space*	0.1261**	*0.0945*	0.2433**
Reference level	Premium coworking space	− 0.0666	0.0747	− 0.2253
*Reception and hospitality*	*Reception but no host*	0.1665***	0.1930**	*0.1150*
Reference level	Reception and active host	0.0444	0.1612	− 0.0142
*Events*	*Sometimes*	0.1586***	0.2604***	*0.181*
Reference level	Often	0.0141	− 0.127	0.0505
*Diversity of tenants*	*Moderate diversity of tenants*	0.1660***	0.3511***	*0.2265*
Reference level	Strong diversity of tenants	0.1579	0.1356	0.4341
*Type of lease contract*	*Short term contract*	0.3440***	0.4199***	0.1749
Reference level	Long term	− 1.0078	− 0.5721	− 0.642
Parameters		26	26	26
Log Likelihood function (LL(β))		− 2122.0810	− 993.1682	− 733.5122
Restricted Log Likelihood function (LL(0))		− 2732.3862	− 1222.7116	− 985.6553
ρ2		0.223	0.188	0.256
ρ2 adjusted		0.220	0.180	0.247
AIC		4296.2	2038.3	1519.0

Note: The italic cells refer to the highest utility per attribute; *significant at p ≤ 0.1 level, **significant at p ≤ 0.05 level, *** significant at p ≤ 0.01 level

**Fig. 3:**
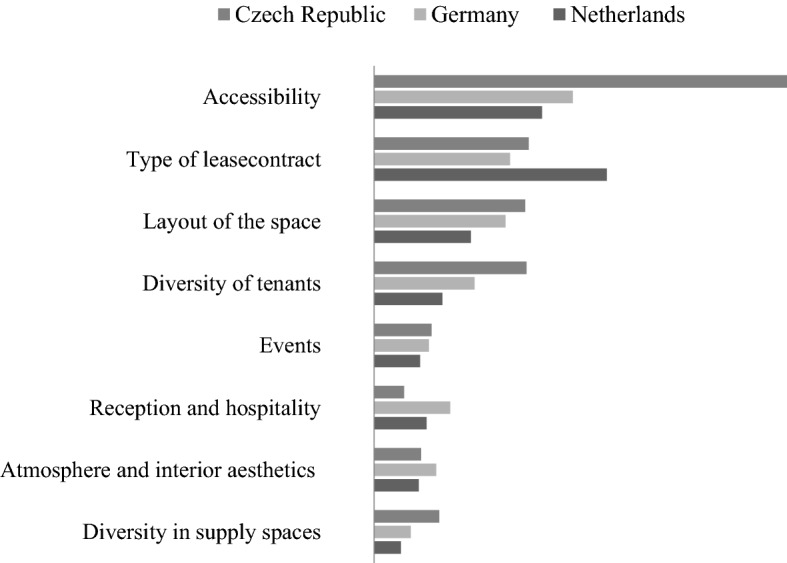
Total utility of attributes per country

Figure 3 shows the utility impacts of all attributes per country, which are computed using the difference between the lowest and highest part-worth utility of the attribute levels. These utilities refer to the importance of each attribute when choosing a coworking space to work at. As can be seen, the accessibility of the location is the most important coworking space attribute for coworkers in the Czech Republic and Germany. For coworkers in the Netherlands, the type of lease contract is more important. When these two main attributes meet one’s preferences, layout and diversity of tenants are also important for choosing between alternatives. Events, reception and hospitality, atmosphere and interior aesthetics and diversity of supply spaces were found to be the least important when choosing between alternative coworking spaces.

Not only the importance of different attributes but also the preferred ‘quality’ level for 3 of the 8 attributes differs between the countries. As 2 of these 3 attributes are at the top of the importance ranking when choosing a specific coworking space, H4 (User preferences for coworking space attributes are consistent across different countries) must be rejected. Concerning accessibility, the MMNL models showed that the part-worth utility of the level accessibility by car and public transport is the highest for the Netherlands and Germany, while in the Czech Republic the probability that coworkers choose a coworking space that is accessible only by public transport is higher. Also, coworkers in the Netherlands and the Czech Republic prefer a homey atmosphere and interior, while coworkers in Germany prefer a more modern interior. An industrial interior is the least preferred by coworkers from the Netherlands and Germany, while a modern interior is the least preferred by coworkers from the Czech Republic. And last, regarding the lease contract, differences were found between the countries as well. Coworkers from the Netherlands and the Czech Republic prefer no lease contract, while coworkers from Germany prefer a short-term lease contract.

No differences were found in preferences related to the other 5 attributes: layout of the space, diversity in supply of spaces, reception and hospitality, events, and diversity of tenants. Coworkers from all the three countries prefer a coworking space with a half-open layout, which consists of open workspaces in combination with spaces for concentration and for formal meetings. Also, no differences were found with regard to the diversity in supply of spaces. Coworkers from all countries prefer a standard coworking space that offers office space with informal meeting areas and event spaces. Moreover, coworkers from all three countries mostly prefer a reception but no host, only sometimes an event (not too often) and a moderate diversity of tenants.

The standard deviations of the random parameters from Table 3 are visible in Table 4. These show that there also still exists unobserved heterogeneity in preferences between coworkers from a specific country for these attributes. High standard deviations imply that even within the country groups, preferences are quite heterogeneous.

**Table 4:** Standard deviations random parameters

		Netherlands (N = 219)	Germany (N = 98)	Czech Republic (N = 79)
Constant	Constant	3.3089***	2.4871***	1.7316***
Accessibility	By car and public transport	0.3426***	0.2482*	0.1986
Atmosphere and interior aesthetics	Industrial	0.25018**	0.4502***	0.2670
Layout of the space	Open layout	0.26087***	0.5934***	0.3606**
Diversity in supply spaces	Basic coworking space	0.02709	0.1586	0.5850***
Reception and hospitality	No reception and no host	0.46216***	0.20860	0.6168***
Events	None	0.14715	0.0942	0.3119*
Diversity of tenants	No diversity of tenants	0.35329***	0.3492***	0.6179***
Type of lease contract	No contract	0.91284***	0.7799***	1.2669***

*significant at p ≤ 0.1 level

**significant at p ≤0.05 level

*** significant at p ≤0.01 level

## Discussion

The analyses have shown several significant differences in preferences and motivations of coworking space users in the three countries that were included in this study. However, regarding motivations, the main attractiveness of coworking spaces in all countries appears to be the vibrant atmosphere and separating work from private life. A bit surprisingly, the novelty-focused motivation of a vibrant, creative atmosphere was followed by typical efficiency-focused real estate arguments of affordability and flexibility, instead of other novelty-focused motivations such as being part of a community, networking and interacting. Regarding efficiency, the Dutch seem to care more about flexibility, the Germans about affordability and the professional appearance for the company, and the Czech about the supportive services of a professional supportive work environment. As Cabral and van Winden (2020) showed with a content analysis of coworking operator websites, operators are well aware of these more individual-level benefits that are sought after. The combination of physical space and co-location is claimed by operators to have an activating effect that pushes workers to bring out the best in themselves, while these operators less often claim to actually create a connection with other people with their spaces (Cabral and van Winden 2020). Nonetheless, the same study showed that they do claim that the community would stimulate individual productivity and growth. These new findings now add that apparently networking and interacting are also a less important motivation to use a coworking space for many users. Both social and work-related interactions and networking are located at the bottom half of the motivation list, only above the professional image gained by using a coworking space. In this sample, the German coworkers did put social interaction with coworkers more often in their top 3 and the Czech coworkers the opportunity to network. As community formation and networking is what distinguishes coworking spaces from other business centres (Weijs-Perrée et al. 2016), the first contribution of this article is that its data raise the question whether the younger, more independent coworking users are really seeking something different than those established companies using regular serviced offices for decades or not.

The second contribution is that the significant differences found in preferences for specific coworking space attributes seem to imply that international chains with uniform coworking space offerings might not satisfy local preferences to the full extent. This suggestion is particularly supported by the fact that especially those attributes coming forward as most important when choosing between different coworking space alternatives, showed significant differences in preferences between countries, namely accessibility and the type of lease contract. The main consistency across countries that came forward is that these two generally important (corporate) real estate choice attributes are indeed the most important ones in all three countries, followed by layout and the diversity of tenants in the coworking space. Only when these aspects are to one’s liking, the more typical coworking space characteristics (events, host, atmosphere, and diversity of spaces) potentially determine the preference for one alternative over the other. Interestingly, for these more typical coworking space attributes, coworkers from all three countries did show similar preferences (atmosphere excluded). They generally seem to prefer a coworking space with a half-open layout, which consists of open workspaces in combination with spaces for concentration and formal meetings. A standard space that offers such spaces, plus a reception, some informal meeting areas and some event space appears to be preferred over a more premium alternative with a host and many events. Also, the diversity of tenants is appreciated, but only at the ‘moderate diversity’ level. Apparently, there is also something as too much diversity in tenants. So, regarding the design/service offer it seems that coworking spaces could be uniform across countries, while for accessibility, contracts and the atmosphere/aesthetics it would be wise to follow more local ‘habits’. This sample suggests that the Czech Republic coworkers care less about accessibility by car and the German coworkers prefer a more modern work environment than the ‘homey’ alternative preferred by the other two countries’ samples. It could however be, that the higher share of students in the Czech sample caused the first difference and the higher share of users from large enterprise in Germany the second one. The sample size did not allow to control for such interaction effects.

The overall consistent preference for the ‘average/standard’ quality levels of these typical coworking space attributes might be explained by the fact that this sample often chose ‘to find affordable accommodation’ as a motivation for using coworking spaces in general. Additional offerings in the higher quality levels could be expected to (and most likely will) be more expensive. Repeating the study with larger samples needs to check whether this is the case for all coworking users or that this sample was specific in that. It does become clear that regarding the top ranked motivation for using coworking spaces—their vibrant and creative atmosphere—the physical support of such an atmosphere (the interior design either being modern, industrial of more like a living room at home) might show different preferences across the countries but is not influencing the choice for a specific coworking space much (see Fig. 3). So, international uniformity in design should not hurt satisfaction of preferences that much. It is especially the accessibility and the contract, that deserve a local flavour to coworking spaces’ offer.

In all three countries the coworking spaces seem to attract young, highly educated users, with a balance between male and female. Coworking spaces do not seem to be a place for full-time work, as the average hours spend there during the week in each country was about 20 h, which is consistent with other research (Kopplin 2020). This sample showed an evident attractiveness to students in the Czech Republic and less so in the Netherlands and Germany, although this could be due to the sampling in the three countries. The trend of more corporates allowing their employees to work in coworking spaces, seems most visible in the German sample, while self-employed workers dominate the Dutch sample. This might be caused by the limitation of this research that the Dutch sample was gathered first (2016) and the German sample later (2018), although the Czech Republic sample which was gathered last (2019) does not show increased corporate use like in Germany. Another explanation could be, that in the Netherlands several large corporates are opening up the ground floor of their own offices for independent workers to create a coworking community, instead of sending their workers to existing coworking spaces.

Overall, the main contribution of this paper is that its findings imply that coworking space user preferences are not that different from those using other office types as assumed, with accessibility and contract being the most important attributes to choose between coworking spaces. It seems that they go through similar decision-making processes.

### Limitations and recommendations for future research

This study did not aim to draw representative samples from coworking spaces in each country and was not able to do so (due to time limits and a limited willingness to participate among coworking providers). Rather, this paper wanted to analyse whether assumptions about internationally consistent user preferences are true, even when looking at a relatively small sample of coworking spaces in only a few countries. Future research should identify significant differences between more countries and based on representative samples, to identify successful coworking space alternatives for more ‘local’ situations. It would be interesting to look at differences between and within continents, as this paper only contains data from within Europe. Larger samples also allow for using more than 8 attributes, because one could randomly show a selection of attributes to respondents instead of showing all respondents all quality levels of all attributes. Additionally, larger samples would allow testing interaction variables in an overall model for all involved countries. Last, large samples would make it possible to do additional analyses in the form of latent class modelling, where groups of respondents with highly similar preferences are identified, and then tested on their individual demographics and other user characteristics to describe more clear target groups for certain coworking space business models.

Future research should also identify why the identified differences exist and whether they are based on nationality, culture or other individual or group characteristics. The standard deviations for the standard utility parameters show that there is still more unobserved heterogeneity within the groups and thus that socio-demographic and other characteristics of individuals need to be included to help explain the choice decisions in more detail. Although some of these were included in this dataset already, the sample size did not allow for testing their prediction of certain preferences.

It would be interesting to include rental prices in future research and do willingness to pay analyses for all attributes, including those that were not that high in the ranking of importance (e.g. a host). This gives providers insights in potential cost savings, that could be invested in more important attributes to attract their target group. In that sense, the clear interest in coworking spaces amongst Czech students suggests to study whether/how students can be an attractive population for coworking operators in other countries, given their limited budget to pay a fee. Besides costs, future research could study the decision-making processes of different types and sizes of companies/entrepreneurs in more detail as well, to provide more insight in sought benefits and compromises that are made in seeking goals.

From a macro-organizational view, Bouncken et al. (2020a) argue that institutional patterns in a coworking space like participation, autonomy and sense of community affect work satisfaction. Future studies should link the underlying space attributes with institutional patterns to show how they rely on the spatial attributes. Additionally, they could make further comparisons on combinations of business models, especially as an increased merge of serviced offices and coworking offices is visible in practice and a rise of new types of corporate coworking initiatives in corporate offices.

Finally, the empirical part of this study was conducted before the beginning of the current Covid 19 pandemic. It cannot be ruled out that user preferences in the countries studied have changed permanently due to the pandemic. However, this question can only be answered by further empirical analyses after the current situation has ended.

## Conclusion and recommendations for practice

This study has shed light on preferences for coworking spaces in different countries. The gathered user preferences can be used as precedents for value propositions of coworking business models that can lead to a higher loyalty of coworking users (Clauss et al. 2019). From a corporate real estate management perspective, the preferences identified can form the basis for sourcing decisions, facilitate location decisions and provide information on which employees feel most comfortable in which form of workplace. For some of the aspects the preferences seem to be consistent and perhaps not that different from preferences for more traditional office types (e.g. a combination of openness and places to concentrate and meet; high importance of accessibility and type of rental contract). Nonetheless, the coworking population is very different from the regular office population, because it is much more diverse. This diversity of users, the variety of facilities and the space design might be additional benefits for corporates through the use of coworking spaces.

Coworking spaces are one of the unique work arrangements that can be used by users all over the world. Large operators are increasingly offering coworking space with a generic standard in their offices worldwide, which however might not serve users from all countries to a similar extent because of the findings here. They provide conformity in standards worldwide for large corporates using these coworking spaces but might not attract local users in all countries this way. Coworking operators can use the identified preferences as guidance on their path from a product-centered to a user-centric environment and help them to find their competitive advantage. Irrespective of country and user type, operators benefit from a good accessibility and can determine the success of their spaces through variable contract periods and a design with half-open layouts. The early, smaller coworking space providers are perhaps still serving a more locally and culturally determined need, which caused the rise of these spaces. The future will tell which users they will continue to attract versus the large global serviced office providers and corporates opening up coworking spaces inside their buildings as well. All will have to meet user preferences in order to compete in this fast-growing, competitive market. The decision to focus on a specific target group can be guided by the results of the study.
